# A Comparison of Knowledge and Skills Related to Up-to-Date Implant Techniques Among Prosthodontists, Periodontists, and Oral Surgeons: A Cross-Sectional Study

**DOI:** 10.7759/cureus.30370

**Published:** 2022-10-17

**Authors:** Bader Fatani, Ebtihal S Almutairi, Hadeel A Almalky, Mazen I Mubarki, Afraa Al-Safadi

**Affiliations:** 1 College of Dentistry, King Saud University, Riyadh, SAU; 2 Pharmacy Department, King Khaled University Hospital, King Saud University Medical City, Riyadh, SAU

**Keywords:** knowledge, techniques, implant, oral surgery, periodontics, prosthodontics

## Abstract

Background

A dental implant is used in the treatment of complete and partial edentulism. Implant application has increased significantly in modern dentistry. The anatomy, medical condition, practitioner knowledge, and surgical technique are key factors that eventually affect the overall outcome of dental implants. For a better treatment outcome for dental implants, adequate multidisciplinary communication, cooperation, and support must be achieved while considering the periodontics, prosthodontics, and oral surgery specialties.

Objective

This study aims to compare the knowledge and skills regarding up-to-date implant techniques among prosthodontists, periodontists, and oral surgeons in Riyadh, Saudi Arabia.

Materials and methods

The data were collected from prosthodontists, periodontists, and oral surgeons working in Riyadh, Saudi Arabia from April 2022 to August 2022. The targeted subjects were residents, specialists, and consultants working in Riyadh, Saudi Arabia.

Results

A total of 181 dentists were surveyed in the current study. A significantly higher proportion of oral surgeons have perceived that the BTI brand (BTI Biotechnology Institute, Álava, Spain) had a high load torque before screw fracture (p = 0.025). Periodontists had significantly higher knowledge levels related to the technique with the lowest marginal bone loss (p = 0.003). Knowledge levels were significantly higher among prosthodontists for the items related to the fact that both early and late implant placement following alveolar ridge placement would have the least changes in periodontal parameters (p = 0.013).

Conclusion

All the targeted specialties exhibited a comparable impression regarding implant techniques and their outcomes. Each specialty had its own aspect of treatment during the placement of dental implants depending on multiple factors. Significant knowledge was observed from each specialty regarding implant brands, techniques, and antibiotic prescriptions for dental implants.

## Introduction

Dental implants were first introduced in the 1970s by Branemark and have become a common alternative treatment for replacing and rehabilitating missing teeth [1]. It is a structure made of alloplastic elements implanted into the oral tissues under the periosteum and within or through the bone to maintain and support a fixed or removable dental prosthesis [2]. The achievement of implant-supported rehabilitation is dependent upon the interaction between the bone and implant surface without the intervention of connective tissue or signs and symptoms of infection or inflammation [3]. A dental implant is used in the treatment of complete and partial edentulism, and its application has increased significantly in modern dentistry [2]. Previous reports demonstrated various techniques in implant placement, including immediate implant placement, which minimizes the number of surgical interventions and the shortening of the total treatment course. However, it has been shown to be associated with esthetic complications [4,5]. Furthermore, it increases the risk of infection and inadequate volume of soft tissue and may present some challenges for some clinicians [4]. Failure rates in the range of 1% to 19% for dental implants were reported in previous studies [1]. The failures rates can be categorized into early and late failures based on the following: first, the period in which the abutment was attached (early failures that occurred earlier to the application of functional loading), and second, late failures that occur following the application of the functional load [1,6-8]. The anatomy, medical condition, practitioner knowledge, and surgical method are key factors that eventually affect the overall result of dental implants [9]. In Saudi Arabia, the only legalized dentists to place an implant are periodontists, oral surgeons, and prosthodontists who had training in the post-grad program. For a better treatment outcome for a dental implant, multidisciplinary, sufficient communication, cooperation, and support must be achieved considering periodontics, prosthodontics, and oral surgery specialties [10,11]. Thus, different implant techniques and their approaches could exhibit different survival rates. In this study, we will compare the knowledge and skills regarding up-to-date implant techniques among prosthodontists, periodontists, and oral surgeons in Riyadh, Saudi Arabia.

## Materials and methods

This research is an observational cross‐sectional study with a sample size of 181 randomized dentists. Permission was acquired from the Research Ethics Committee of King Khaled University Hospital (KKUH). The data were collected from residents, specialists, and consultants working in the prosthodontic, periodontics, and oral surgery specialties in Riyadh, Saudi Arabia. All information regarding the research questionnaire was explained, and the consent form was approved by each participant. The study was conducted from April 2022 to August 2022. The targeted subjects were male or female residents, specialists, and consultants who work in the prosthodontic, periodontics, or oral surgery specialties in Riyadh, Saudi Arabia. Those who are not residents, specialists, or consultants, or those who are not working in prosthodontic, periodontics, or oral surgery specialties were excluded. The questionnaire contained 34 questions that were reviewed by two specialized reviewers before submitting the survey to the targeted participants. The questionnaire was distributed to multiple centers in Riyadh. The data collection was carried out by two methods to reach the desired sample size. First, a simple random sampling of (152) electronic questionnaires (also referred to as ‘Monkey Survey or Google Form’) was distributed to residents, specialists, and consultants through e-mail and other social media networks to reach a various number of dentists in Riyadh City. Second, a random sampling through face-to-face interviews (29 in total) was conducted with the residents, specialists, and consultants working at King Saud University. The minimum sample size for this study has been decided according to Swinscow. The z-value for the selected level of confidence was 1.96 with an estimated prevalence of prosthodontists, periodontists, and oral surgeons in Riyadh, Saudi Arabia. The maximum acceptable error was 0.05. Therefore, the calculated minimum sample size was 172. To prevent any prejudice or bias in the outcome of the results, the study was ensured to be supervised by an individual of different specialty other than the targeted specialties.

## Results

Demographic and practice-related characteristics

In the current study, we included the responses of 181 dentists. The distribution of participants was almost equal in terms of their specialties, where periodontists, oral surgeons, and prosthodontists represented 37.6%, 34.8%, and 27.6% of the sample, respectively. Similarly, the distribution of residents, specialists, and consultants was 28.7%, 34.3%, and 37.0%, respectively. More than one-third of dentists have performed >30 dental implants, and more than half of them (58.0%) have ever experienced failed dental implants. The majority of participants (92.8%) recommended the prescription of analgesics after dental implants, whereas 66.9% of them recommended antibiotics prescription (Table 1).

**Table 1:** Demographic and practice-related characteristics

Parameter	Category	N (%)
Specialty	Periodontics	68 (37.6%)
	Prosthodontics	50 (27.6%)
	Oral surgery	63 (34.8%)
What is your current educational level?	Resident	52 (28.7%)
	Specialist	62 (34.3%)
	Consultant	67 (37.0%)
The number of dental implants performed	1 to 10	41 (22.7%)
	10 to 20	40 (22.1%)
	20 to 30	28 (15.5%)
	>30	72 (39.8%)
Have ever experienced any failed dental implants	Yes	105 (58.0%)
Recommend the prescription of antibiotics after each dental implant	Yes	121 (66.9%)
Recommend the use of analgesics after each dental implant	Yes	168 (92.8%)

Knowledge and skills of the overall sample

Based on the participant's responses, the BIOMET 3i® (Palm Beach Gardens, Florida) brand was the most commonly agreed brand that has a high load torque before screw fracture (47.5%). Clinical parameters were thought to be the most common risk factors, which would lead to a subsequent late failure of a dental implant (51.4%). From the dentists’ points of view, conventional immediate dental implant placement was the most common technique that has the lowest marginal bone loss (51.4%) and the best aesthetic outcome of soft tissue around the implant (42.0%). Additionally, late implant placement following alveolar ridge preservation has the most significant changes in the midfacial mucosal margin (46.4%), the highest pink/white esthetic scores (45.9%), and the least changes in periodontal parameters (50.8%). The remaining responses to other questions are listed in Table 2.

**Table 2:** Participants’ responses regarding their knowledge and skills toward up-to-date implant techniques BIOMET 3i®: Palm Beach Gardens, Florida; BTI®: BTI Biotechnology Institute, Álava, Spain

Parameter	Category*	N (%)*
Regarding dental implant brands, which one of these would you suggest having a high load torque before screw fracture?	BIOMET 3i®	86 (47.5%)
	BTI®	35 (19.3%)
	Dentium®	40 (22.1%)
	Megagen®	20 (11.0%)
From your point of view, what is the most common risk factor related to a late failure of a dental implant?	Patient history	44 (24.3%)
	Clinical parameters	93 (51.4%)
	Decisions made by the doctor	44 (24.3%)
From your point of view, which one of these has lower marginal bone loss?	Conventional immediate dental implant placement	93 (51.4%)
	Socket shield	58 (32.0%)
	No difference	30 (16.6%)
From your point of view, which one of these techniques has the more aesthetic outcome of soft tissue around the implant?	Conventional immediate dental implant placement	76 (42.0%)
	Socket shield	71 (39.2%)
	No difference	34 (18.8%)
Which implant placement technique would you recommend for a more successful outcome?	Free-hand implant placement	51 (28.2%)
	Guided implant placement	66 (36.5%)
	Both	64 (35.4%)
From your point of view, which Implant placement technique for non-molar extraction has more changes in the midfacial mucosal margin?	Early implantation	65 (35.9%)
	Late implant placement following alveolar ridge presentation	84 (46.4%)
	No difference	32 (17.7%)
From your point of view, Which Implant placement technique for non-molar extraction has more Pink/white esthetic scores?	Early implantation	68 (37.6%)
	Late implant placement following alveolar ridge presentation	83 (45.9%)
	No difference	30 (16.6%)
Regarding having less changes in periodontal parameters, what is your preferred Implant placement technique for non-molar extraction?	Early implantation	51 (28.2%)
	Late implant placement following alveolar ridge presentation	92 (50.8%)
	All techniques will be the same result	38 (21.0%)
From your point of view, when trying to reduce the failure rate of the implant in a non-compromised patient, it will be reduced when you?	Give the patients just a preoperative antibiotic	56 (30.9%)
	Give the patients both pre and postoperative antibiotics	69 (38.1%)
	All of them will reduce the failure rate of implants with no difference	56 (30.9%)
From your point of view, which type of graft material has superior biomechanical osseointegration of titanium implants after guided bone regeneration (GBR)?	Allograft	88 (48.6%)
	Hydroxyapatite	35 (19.3%)
	Calcium sulfate	17 (9.4%)
	Deproteinized bovine bone graft	41 (22.7%)
From your point of view, what do you prefer to get better clinical and radiographical bone healing?	Immediate implants with or without bone graft	43 (23.8%)
	Delay implants with or without bone graft	138 (76.2%)
Which of these graft materials would you think will increase the implant stability and marginal bone levels after immediate placement of the implant?	Xenograft bone material	38 (21.0%)
	Freeze-dried bone allograft	78 (43.1%)
	There is no difference between them for more implant stability	65 (35.9%)
From your point of view, what is the most effective technique for peri-implant keratinized mucosa width augmentation?	Free periosteal graft	76 (42.0%)
	Free connective tissue graft	57 (31.5%)
	Apically positioned flap in combination with Free gingival graft	48 (26.5%)
In terms of mucosal thickness (MT) gain. from your point of view, what do you think is the most superior technique in terms of success?	Xenogeneic collagen matrix	16 (8.9%)
	Bilaminar techniques in combination with a connective tissue graft	79 (43.9%)
	Bilaminar techniques in combination with acellular dermal matrix	85 (47.2%)
Regarding digital dentistry, do you think yourself?	Poorly informed	23 (12.7%)
	Adequately informed	68 (37.6%)
	Well informed	66 (36.5%)
	Very well informed	24 (13.3%)
Regarding the digital scans with intraoral scanners for dental implants, from your point of view, their accuracy is most dependent on?	Optical scanning devices	35 (19.3%)
	Software used	49 (27.1%)
	Both	97 (53.6%)
Regarding the role of intraoral scan bodies (ISBs) in digital implant impressions, do you think yourself?	Poorly informed	37 (20.4%)
	Adequately informed	71 (39.2%)
	Well informed	58 (32.0%)
	Very well informed	15 (8.3%)
From your point of view, can intraoral scanners for dental implants replace the conventional impression in the future?	Yes	137 (75.7%)
From your point of view, what is the most technical complication associated with dental implants?	Implant body fracture	51 (28.2%)
	Abutment screw loosening or fracture	50 (27.6%)
	Abutment and superstructure fracture	80 (44.2%)
Regarding head and neck cancer patients, would you recommend the placement of dental implants during ablative surgery in head and neck cancer patients?	Yes	148 (81.8%)
When evaluating the time and costs involved with the diagnostic and planning procedures for computer-assisted implant planning and surgery would you suggest?	Time and costs are lower than for non-computer-assisted	42 (23.2%)
	Time and costs are higher than for non-computer-assisted	130 (71.8%)
	Time and costs are equal for non-computer-assisted	9 (5.0%)
From your experience, do you think the use of NSAIDs, and selective serotonin reuptake inhibitors (SSRIs) would have a significant effect on implant failure rate?	Yes	108 (59.7%)
From your point of view, is there a need to increase the knowledge and awareness regarding the potential risk factors that could impact implant failures for the dentist who is practicing dental implants?	Yes	165 (91.2%)
If yes, what is the best way to achieve it?	Regular assessment of the theoretical and practical knowledge of implant dentistry	73 (40.3%)
	Continuous dental education programs and workshops	105 (58.0%)
	Not applicable	3 (1.7%)
Based on your experience, do you think Immediate loading implants have a higher failure rate than conventional-loading implants?	Yes	164 (90.6%)
Do you usually give prophylactic antibiotics prior to implant placement?	Yes	146 (80.7%)
Do you think postoperative analgesics are important in managing patients’ pain and increasing satisfaction?	Yes	173 (95.6%)

Knowledge and skills based on participants’ specialty

A significantly higher proportion of oral surgeons have correctly agreed that the BTI brand had a high load torque before screw fracture (28.6% of oral surgeons versus 12.0% of prosthodontists and 16.2% of periodontists, p = 0.025). Periodontists had a significantly higher knowledge level in distinct items as indicated by their answers to items related to the technique, which has the lowest marginal bone loss (47.1% of periodontists versus 24.0% of prosthodontists and 22.2% of oral surgeons, p = 0.003) and the technique that induces the best aesthetic outcome of soft tissues (52.9% of periodontists versus 32.0% of prosthodontists and 30.2% of oral surgeons, p = 0.013), as well as the fact that time and costs are higher for non-computer-assisted implant planning and surgery than computer-assisted procedures (85.3% of periodontists versus 48.0% of prosthodontists and 76.2% of oral surgeons, p < 0.001). Knowledge levels were significantly higher among prosthodontists for the items related to the fact that both early and late implant placement following alveolar ridge placement would have the least changes in periodontal parameters (32.0% of prosthodontists versus 19.1% of periodontists and 14.3% of oral surgeons, p = 0.013) and that the use of non-steroidal anti-inflammatory drugs (NSAIDs) and selective serotonin reuptake inhibitors would have a significant effect on implant failure rate (78.0% of prosthodontists versus 54.4% of periodontists and 50.8% of oral surgeons, p = 0.007). There were also significant differences among different specialties in terms of their perceptions regarding the most superior technique that induces success in mucosal thickness gain (p = 0.016) and self-perceptions regarding digital dentistry (p = 0.004, Table 3).

**Table 3:** Participants’ responses regarding their knowledge and skills stratified by participants’ specialty BIOMET 3i®: Palm Beach Gardens, Florida; BTI®: BTI Biotechnology Institute, Álava, Spain

Parameter	Category	Periodontics, N = 68	Prosthodontics, N = 50	Oral surgery, N = 63	p-value
Regarding dental implant brands, which one of these would you suggest having a high load torque before screw fracture?	BIOMET 3i®	41 (60.3%)	20 (40.0%)	25 (39.7%)	0.025
	BTI®	11 (16.2%)	6 (12.0%)	18 (28.6%)	
	Dentium®	9 (13.2%)	17 (34.0%)	14 (22.2%)	
	Megagen®	7 (10.3%)	7 (14.0%)	6 (9.5%)	
From your point of view, what is the most common risk factor related to a late failure of a dental implant?	Patient history	15 (22.1%)	18 (36.0%)	11 (17.5%)	0.089
	Clinical parameters	40 (58.8%)	21 (42.0%)	32 (50.8%)	
	Decisions made by the doctor	13 (19.1%)	11 (22.0%)	20 (31.7%)	
From your point of view, which one of these has lower marginal bone loss?	Conventional immediate dental implant placement	25 (36.8%)	26 (52.0%)	42 (66.7%)	0.003
	Socket shield	32 (47.1%)	12 (24.0%)	14 (22.2%)	
	No difference	11 (16.2%)	12 (24.0%)	7 (11.1%)	
From your point of view, which one of these techniques has the more aesthetic outcome of soft tissue around the implant?	Conventional immediate dental implant placement	20 (29.4%)	21 (42.0%)	35 (55.6%)	0.013
	Socket shield	36 (52.9%)	16 (32.0%)	19 (30.2%)	
	No difference	12 (17.6%)	13 (26.0%)	9 (14.3%)	
Which implant placement technique would you recommend for a more successful outcome?	Free-hand implant placement	15 (22.1%)	20 (40.0%)	16 (25.4%)	0.163
	Guided implant placement	24 (35.3%)	15 (30.0%)	27 (42.9%)	
	Both	29 (42.6%)	15 (30.0%)	20 (31.7%)	
From your point of view, which implant placement technique for non-molar extraction has more changes in the midfacial mucosal margin?	Early implantation (EP)	27 (39.7%)	11 (22.0%)	27 (42.9%)	0.097
	Late implant placement following alveolar ridge presentation (LP/ARP)	29 (42.6%)	26 (52.0%)	29 (46.0%)	
	No difference	12 (17.6%)	13 (26.0%)	7 (11.1%)	
From your point of view, Which Implant placement technique for non-molar extraction has more Pink/white esthetic scores?	Early implantation (EP)	25 (36.8%)	15 (30.0%)	28 (44.4%)	0.223
	Late implant placement following alveolar ridge preservation (LP/ARP)	34 (50.0%)	22 (44.0%)	27 (42.9%)	
	No difference	9 (13.2%)	13 (26.0%)	8 (12.7%)	
Regarding having less changes in periodontal parameters, what is your preferred Implant placement technique for non-molar extraction?	Early implantation (EP)	9 (13.2%)	12 (24.0%)	30 (47.6%)	<0.001
	Late implant placement following alveolar ridge preservation (LP/ARP)	46 (67.6%)	22 (44.0%)	24 (38.1%)	
	All techniques will be the same result	13 (19.1%)	16 (32.0%)	9 (14.3%)	
From your point of view, when trying to reduce the failure rate of the implant in a non-compromised patient, it will be reduced when you?	Give the patients just a preoperative antibiotic	28 (41.2%)	16 (32.0%)	12 (19.0%)	0.084
	Give the patients both pre and postoperative antibiotic	23 (33.8%)	20 (40.0%)	26 (41.3%)	
	All of them will reduce the failure rate of implants to no difference	17 (25.0%)	14 (28.0%)	25 (39.7%)	
From your point of view, which type of graft material has superior biomechanical osseointegration of titanium implants after guided bone regeneration (GBR)?	Allograft (ALG)	42 (61.8%)	16 (32.0%)	30 (47.6%)	0.069
	Hydroxyapatite (HA)	11 (16.2%)	11 (22.0%)	13 (20.6%)	
	Calcium sulfate (CP)	4 (5.9%)	8 (16.0%)	5 (7.9%)	
	Deproteinized bovine bone graft (DPBB)	11 (16.2%)	15 (30.0%)	15 (23.8%)	
From your point of view, what do you prefer to get better clinical and radiographical bone healing?	Immediate implants with or without bone graft	12 (17.6%)	10 (20.0%)	21 (33.3%)	0.083
	Delay implants with or without bone graft	56 (82.4%)	40 (80.0%)	42 (66.7%)	
Which of these graft materials would you think will increase the implant stability and marginal bone levels after immediate placement of the implant?	Xenograft bone material	11 (16.2%)	12 (24.0%)	15 (23.8%)	0.746
	Freeze-dried bone allograft	32 (47.1%)	19 (38.0%)	27 (42.9%)	
	There is no difference between them for more implant stability	25 (36.8%)	19 (38.0%)	21 (33.3%)	
From your point of view, what is the most effective technique for peri-implant keratinized mucosa width augmentation?	Free periosteal graft (FPG)	30 (44.1%)	20 (40.0%)	26 (41.3%)	0.823
	Free connective tissue graft (FCG)	23 (33.8%)	14 (28.0%)	20 (31.7%)	
	Apically positioned flap (APF) in combination with Free gingival graft (FGG)	15 (22.1%)	16 (32.0%)	17 (27.0%)	
In terms of mucosal thickness (MT) gain. from your point of view, what do you think is the most superior technique in terms of success?	Xenogeneic collagen matrix	4 (5.9%)	7 (14.3%)	5 (7.9%)	0.016
	Bilaminar techniques in combination with a connective tissue graft	22 (32.4%)	21 (42.9%)	36 (57.1%)	
	Bilaminar techniques in combination with acellular dermal matrix	42 (61.8%)	21 (42.9%)	22 (34.9%)	
Regarding Digital dentistry, do you think yourself?	Poorly informed	4 (5.9%)	14 (28.0%)	5 (7.9%)	0.004
	Adequately informed	23 (33.8%)	21 (42.0%)	24 (38.1%)	
	Well informed	31 (45.6%)	11 (22.0%)	24 (38.1%)	
	Very well informed	10 (14.7%)	4 (8.0%)	10 (15.9%)	
Regarding the digital scans with intraoral scanners for dental implants, from your point of view, their accuracy is most dependent on?	Optical scanning devices	9 (13.2%)	14 (28.0%)	12 (19.0%)	0.127
	Software used	15 (22.1%)	15 (30.0%)	19 (30.2%)	
	Both	44 (64.7%)	21 (42.0%)	32 (50.8%)	
Regarding the role of intraoral scan bodies (ISBs) in digital implant impressions, do you think yourself?	Poorly informed	10 (14.7%)	13 (26.0%)	14 (22.2%)	0.509
	Adequately informed	26 (38.2%)	20 (40.0%)	25 (39.7%)	
	Well informed	23 (33.8%)	14 (28.0%)	21 (33.3%)	
	Very well informed	9 (13.2%)	3 (6.0%)	3 (4.8%)	
From your point of view, can intraoral scanners for dental implants replace the conventional impression in the future?	Yes	53 (77.9%)	38 (76.0%)	46 (73.0%)	0.805
From your point of view, what is the most technical complication associated with dental implants?	Implant body fracture	18 (26.5%)	19 (38.0%)	14 (22.2%)	0.088
	Abutment screw loosening or fracture	20 (29.4%)	7 (14.0%)	23 (36.5%)	
	Abutment and superstructure fracture	30 (44.1%)	24 (48.0%)	26 (41.3%)	
Regarding head and neck cancer patients, would you recommend the placement of dental implants during ablative surgery in head and neck cancer patients?	Yes	52 (76.5%)	40 (80.0%)	56 (88.9%)	0.171
When evaluating the time and costs involved with the diagnostic and planning procedures for computer-assisted implant planning and surgery would you suggest?	Time and costs are lower than for non-computer-assisted	9 (13.2%)	20 (40.0%)	13 (20.6%)	<0.001
	Time and costs are higher than for non-computer-assisted	58 (85.3%)	24 (48.0%)	48 (76.2%)	
	Time and costs are equal for non-computer-assisted	1 (1.5%)	6 (12.0%)	2 (3.2%)	
From your experience, do you think the use of NSAIDs, and selective serotonin reuptake inhibitors (SSRIs) would have a significant effect on implant failure rate?	Yes	37 (54.4%)	39 (78.0%)	32 (50.8%)	0.007
From your point of view, is there a need to increase the knowledge and awareness regarding the potential risk factors that could impact implant failures for the dentist who is practicing dental implants?	Yes	61 (89.7%)	45 (90.0%)	59 (93.7%)	0.718
If yes, what is the best way to achieve it?	Regular assessment of the theoretical and practical knowledge of implant dentistry	26 (38.2%)	24 (48.0%)	23 (36.5%)	0.725
	Continuous dental educational programs and workshops	41 (60.3%)	25 (50.0%)	39 (61.9%)	
	Not applicable	1 (1.5%)	1 (2.0%)	1 (1.6%)	
Based on your experience, do you think Immediate loading implants have a higher failure rate than conventional loading implants?	Yes	59 (86.8%)	45 (90.0%)	60 (95.2%)	0.248
Do you usually give prophylactic antibiotics prior to implant placement?	Yes	52 (76.5%)	39 (78.0%)	55 (87.3%)	0.250
Do you think postoperative analgesics are important in managing patients’ pain and increasing satisfaction?	Yes	67 (98.5%)	45 (90.0%)	61 (96.8%)	0.094

Knowledge and skills based on participants’ educational level

A lower proportion of consultants provided adequate responses regarding the best technique, which has lower marginal bone loss (25.4% among consultants versus 33.9% among specialists and 38.5% among residents, p = 0.043). However, a significantly higher proportion of consultants considered themselves as very well informed about the role of intraoral scan bodies in digital implant impressions compared to other educational levels (17.9% among consultants versus 3.2% among specialists and 1.9% among residents, p = 0.033, Table 4). No other significant differences in knowledge and skills were noted among different educational levels.

**Table 4:** Participants’ responses regarding their knowledge and skills stratified by participants’ educational level BIOMET 3i®: Palm Beach Gardens, Florida; BTI®: BTI Biotechnology Institute, Álava, Spain

Parameter	Category	Resident, N = 52	Specialist, N = 62	Consultant, N = 67	p-value
Regarding dental implant brands, which one of these would you suggest having a high load torque before screw fracture?	BIOMET 3i®	25 (48.1%)	29 (46.8%)	32 (47.8%)	0.482
	BTI®	14 (26.9%)	12 (19.4%)	9 (13.4%)	
	Dentium®	7 (13.5%)	15 (24.2%)	18 (26.9%)	
	Megagen®	6 (11.5%)	6 (9.7%)	8 (11.9%)	
From your point of view, what is the most common risk factor related to a late failure of a dental implant?	Patient history	14 (26.9%)	14 (22.6%)	16 (23.9%)	0.946
	Clinical parameters	26 (50.0%)	34 (54.8%)	33 (49.3%)	
	Decisions made by the doctor	12 (23.1%)	14 (22.6%)	18 (26.9%)	
From your point of view, which one of these has lower marginal bone loss?	Conventional immediate dental implant placement	28 (53.8%)	25 (40.3%)	40 (59.7%)	0.043
	Socket shield	20 (38.5%)	21 (33.9%)	17 (25.4%)	
	No difference	4 (7.7%)	16 (25.8%)	10 (14.9%)	
From your point of view, which one of these techniques has the more aesthetic outcome of soft tissue around the implant?	Conventional immediate dental implant placement	20 (38.5%)	26 (41.9%)	30 (44.8%)	0.218
	Socket shield	26 (50.0%)	20 (32.3%)	25 (37.3%)	
	No difference	6 (11.5%)	16 (25.8%)	12 (17.9%)	
Which implant placement technique would you recommend for a more successful outcome?	Free-hand implant placement	12 (23.1%)	21 (33.9%)	18 (26.9%)	0.677
	Guided implant placement	22 (42.3%)	21 (33.9%)	23 (34.3%)	
	Both	18 (34.6%)	20 (32.3%)	26 (38.8%)	
From your point of view, which Implant placement technique for non-molar extraction has more changes of the midfacial mucosal margin?	Early implantation (EP)	22 (42.3%)	23 (37.1%)	20 (29.9%)	0.291
	Late implant placement following alveolar ridge preservation (LP/ARP)	25 (48.1%)	25 (40.3%)	34 (50.7%)	
	No difference	5 (9.6%)	14 (22.6%)	13 (19.4%)	
From your point of view, Which Implant placement technique for non-molar extraction has more Pink/white esthetic scores?	Early implantation (EP)	22 (42.3%)	25 (40.3%)	21 (31.3%)	0.223
	Late implant placement following alveolar ridge preservation (LP/ARP)	26 (50.0%)	24 (38.7%)	33 (49.3%)	
	No difference	4 (7.7%)	13 (21.0%)	13 (19.4%)	
Regarding having less changes in periodontal parameters, what is your preferred Implant placement technique for non-molar extraction?	Early implantation (EP)	20 (38.5%)	15 (24.2%)	16 (23.9%)	0.208
	Late implant placement following alveolar ridge presentation (LP/ARP)	26 (50.0%)	32 (51.6%)	34 (50.7%)	
	All techniques will be the same result	6 (11.5%)	15 (24.2%)	17 (25.4%)	
From your point of view, when trying to reduce the failure rate of the implant in a non-compromised patient, it will be reduced when you?	Give the patients just a preoperative antibiotic	14 (26.9%)	20 (32.3%)	22 (32.8%)	0.843
	Give the patients both pre and postoperative antibiotic	19 (36.5%)	23 (37.1%)	27 (40.3%)	
	All of them will reduce the failure rate of implants with no difference	19 (36.5%)	19 (30.6%)	18 (26.9%)	
From your point of view, which type of graft material has superior biomechanical osseointegration of titanium implants after guided bone regeneration (GBR)?	Allograft (ALG)	28 (53.8%)	26 (41.9%)	34 (50.7%)	0.568
	Hydroxyapatite (HA)	12 (23.1%)	12 (19.4%)	11 (16.4%)	
	Calcium sulfate (CP)	2 (3.8%)	8 (12.9%)	7 (10.4%)	
	Deproteinized bovine bone graft (DPBB)	10 (19.2%)	16 (25.8%)	15 (22.4%)	
From your point of view, what do you prefer to get better clinical and radiographical bone healing?	Immediate implants with or without bone graft	16 (30.8%)	13 (21.0%)	14 (20.9%)	0.371
	Delay implants with or without bone graft	36 (69.2%)	49 (79.0%)	53 (79.1%)	
Which of these graft materials would you think will increase the implant stability and marginal bone levels after immediate placement of the implant?	Xenograft bone material	13 (25.0%)	12 (19.4%)	13 (19.4%)	0.560
	Freeze-dried bone allograft	25 (48.1%)	27 (43.5%)	26 (38.8%)	
	There is no difference between them for more implant stability	14 (26.9%)	23 (37.1%)	28 (41.8%)	
From your point of view, what is the most effective technique for peri-implant keratinized mucosa width augmentation?	Free periosteal graft (FPG)	22 (42.3%)	28 (45.2%)	26 (38.8%)	0.729
	Free connective tissue graft (FCG)	14 (26.9%)	18 (29.0%)	25 (37.3%)	
	Apically positioned flap (APF) in combination with Free gingival graft (FGG)	16 (30.8%)	16 (25.8%)	16 (23.9%)	
In terms of mucosal thickness (MT) gain. from your point of view, what do you think is the most superior technique in terms of success?	Xenogeneic collagen matrix	3 (5.8%)	7 (11.3%)	6 (9.1%)	0.588
	Bilaminar techniques in combination with a connective tissue graft	27 (51.9%)	23 (37.1%)	29 (43.9%)	
	Bilaminar techniques in combination with acellular dermal matrix	22 (42.3%)	32 (51.6%)	31 (47.0%)	
Regarding Digital dentistry, do you think yourself?	Poorly informed	6 (11.5%)	7 (11.3%)	10 (14.9%)	0.134
	Adequately informed	19 (36.5%)	27 (43.5%)	22 (32.8%)	
	Well informed	23 (44.2%)	23 (37.1%)	20 (29.9%)	
	Very well informed	4 (7.7%)	5 (8.1%)	15 (22.4%)	
Regarding the digital scans with intraoral scanners for dental implants, from your point of view, their accuracy is most dependent on?	Optical scanning devices	7 (13.5%)	15 (24.2%)	13 (19.4%)	0.429
	Software used	15 (28.8%)	19 (30.6%)	15 (22.4%)	
	Both	30 (57.7%)	28 (45.2%)	39 (58.2%)	
Regarding the role of intraoral scan bodies (ISBs) in digital implant impressions, do you think yourself?	Poorly informed	14 (26.9%)	13 (21.0%)	10 (14.9%)	0.033
	Adequately informed	22 (42.3%)	24 (38.7%)	25 (37.3%)	
	Well informed	15 (28.8%)	23 (37.1%)	20 (29.9%)	
	Very well informed	1 (1.9%)	2 (3.2%)	12 (17.9%)	
From your point of view, can intraoral scanners for dental implants replace the conventional impression in the future?	Yes	42 (80.8%)	41 (66.1%)	54 (80.6%)	0.096
From your point of view, what is the most technical complication associated with dental implants?	Implant body fracture	12 (23.1%)	18 (29.0%)	21 (31.3%)	0.335
	Abutment screw loosening or fracture	18 (34.6%)	12 (19.4%)	20 (29.9%)	
	Abutment and superstructure fracture	22 (42.3%)	32 (51.6%)	26 (38.8%)	
Regarding head and neck cancer patients, would you recommend the placement of dental implants during ablative surgery in head and neck cancer patients?	Yes	43 (82.7%)	49 (79.0%)	56 (83.6%)	0.783
When evaluating the time and costs involved with the diagnostic and planning procedures for computer-assisted implant planning and surgery, would you suggest?	Time and costs are lower than for non-computer-assisted	11 (21.2%)	18 (29.0%)	13 (19.4%)	0.473
	Time and costs are higher than for non-computer-assisted	40 (76.9%)	41 (66.1%)	49 (73.1%)	
	Time and costs are equal for non-computer-assisted	1 (1.9%)	3 (4.8%)	5 (7.5%)	
From your experience, do you think the use of NSAIDs, and selective serotonin reuptake inhibitors (SSRIs) would have a significant effect on implant failure rate?	Yes	25 (48.1%)	39 (62.9%)	44 (65.7%)	0.124
From your point of view, is there a need to increase the knowledge and awareness regarding the potential risk factors that could impact implant failures for the dentist who is practicing dental implants?	Yes	49 (94.2%)	56 (90.3%)	60 (89.6%)	0.681
If yes, what is the best way to achieve it?	Regular assessment of the theoretical and practical knowledge of implant dentistry	18 (34.6%)	32 (51.6%)	23 (34.3%)	0.136
	Continuous dental educational programs and workshops	33 (63.5%)	30 (48.4%)	42 (62.7%)	
	Not applicable	1 (1.9%)	0 (0.0%)	2 (3.0%)	
Based on your experience, do you think Immediate loading implants have a higher failure rate than conventional loading implants?	Yes	48 (92.3%)	55 (88.7%)	61 (91.0%)	0.855
Do you usually give prophylactic antibiotics prior to implant placement?	Yes	39 (75.0%)	55 (88.7%)	52 (77.6%)	0.132
Do you think postoperative analgesics are important in managing patients’ pain and increasing satisfaction?	Yes	51 (98.1%)	58 (93.5%)	64 (95.5%)	0.537

## Discussion

Several studies discussed implant techniques and their outcomes; these studies illustrated that different implant techniques and their approaches could exhibit different survival rates. According to a previous study, which was done in the USA and aimed to analyze the evidence regarding the efficacy of soft tissue augmentation procedures, the results showed that an apically positioned flap (APF) in combination with a free gingival graft (FGG) is the most effective technique for peri-implant keratinized mucosa width augmentation [12]. However, in our study, all the targeted specialties considered that a free periosteal graft (FPG) is the most effective technique for peri-implant keratinized mucosa width augmentation with periodontists (44.1%) being the most common specialty to use this technique. The same previous study also illustrated that bilaminar techniques in combination with connective tissue grafts are the most superior technique in terms of mucosal thickness (MT) gain [12]. In correlation to our study, oral surgeons (57.1%) had a similar impression regarding this technique. However, most periodontists (61.8%) recommended the use of bilaminar techniques in combination with an acellular dermal matrix for better mucosal thickness (MT) gain. According to Sawase et al., the accuracy of digital scans with intraoral scanners was dependent on the optical scanning devices and software used [13]. Our study showed a similar impression from all specialties regarding the use of intraoral scanners, with periodontists (64.7%) being the most relatable specialty. A previous study by In't Veld M. et al aimed to identify the treatment outcome of immediate placement and loading of dental implants in the edentulous mandible of overdentures in head and neck cancer patients. This study demonstrated a high survival rate of dental implants placed during ablative surgery in head and neck cancer patients [14]. Our study showed that most specialties recommend the placement of dental implants during ablative surgery in head and neck cancer patients with oral surgeons (88.9%) being the most approved specialty. The majority of periodontists (85.3%) suggested that the time and costs involved with the diagnostic and planning procedures for computer-assisted implant planning and surgery are higher than for non-computer-assisted, which demonstrates a similar result to a previous study, which was done by Graf T et al. [10].

A recent study has shown that BTI® has more high load torque before screw fracture. However, in our study, most of the specialties considered that BIOMET 3i® has more load torque before screw fracture than the other brands [3]. The Velasco et al. study found that the socket-shield technique compared to the conventional technique has lower marginal bone loss and higher esthetic scores [15]. In our study, according to lower marginal bone loss, most periodontists (47.1%) agreed with the previous study and most of the oral surgeons (66.7%) and prosthodontists (52.0%) saw that the conventional technique has a lower bone marginal bone loss. For a higher esthetic score, most of the periodontists (52.9%) saw that the socket shield has a higher score compared to oral surgeons (30.2%) and prosthodontists (32.0%). For a more successful outcome, most of the oral surgeons (42.9%) recommended using guided implant placement compared to periodontists (42.6%) who recommended using both techniques, guided and freehand implant placement. However, Abdelhay et al. recommended a guided implant placement approach for a more successful outcome [9]. According to Lim et al., there is no difference in implant placement technique for non-molar extraction, which has more changes in a midfacial mucosal margin between early implantation (EP) and late implant placement following alveolar ridge presentation (LP/ARP) [3]. However, most of the specialties considered that late implant placement following alveolar ridge presentation has more changes in the discussed case [16]. In addition, the previous study showed that there is no difference between early implantation and late implant placement following alveolar ridge preservation in the pink/white esthetic scores [16]. However, in our study, most of the specialties (45.9%) considered that LP/ARP is associated with a more pink/white esthetic score. The same previous study found that EP and LP/ARP had no difference in the periodontal parameters [16]. However, in our study, 50.8% of the specialties considered that LP/ARP has fewer changes in periodontal parameters than EP. Regarding the use of antibiotics to reduce implant failure, some of the participants (30.9%) recommended giving patients just a single preoperative antibiotic, and the same study considered that there are no differences between them to reduce the failure rate in dental implants. In addition, 38.1% of our participants considered that it is better to give the patients both pre and postoperative antibiotics. In the Esposito M et al. and Roca-Millan E et al. study., they considered that there is no difference between a single preoperative antibiotic or both pre and postoperative antibiotics [17,18]. According to Gunes et al., they found that when using biomechanical osseointegration of titanium implants after guided bone regeneration (GBR) with a hydroxyapatite graft, deproteinized bovine bone graft, human-derived allograft, and calcium sulfate bone graft, none of the grafts used were distinctly superior to any of the others [19]. However, in our study, most of the specialties considered that allograft has superior biomechanical osseointegration compared to other types. A previous study that was done by Singh et al. aimed to compare bone healing in immediate implant placement and delayed implant placement. This study showed that delayed Implant placement had better clinical and radiographical bone healing than immediate implant placement [20]. However, in our study, most of the specialties (76.2%) considered that delayed implant placement had better bone healing than immediate implant placement. According to bone graft materials, 47.1% of periodontists and 42.9% of oral surgeons considered that freeze-dried bone allografts will increase the implant stability and marginal bone levels after immediate placement of the implant more than xenograft bone material. In addition, there are prosthodontists (38.0%) who agreed with the other specialties, and 38.0% of them considered no differences between the two materials for more stability. Conversely, the Jalaluddin M et al. study demonstrated that both bone grafting materials showed improvement in marginal bone levels of implant stability after immediate placement of an implant [21]. A previous study done by Dutta SR et al. showed that there is a need to increase the knowledge and awareness regarding the potential risk factors that could impact implant failures in those who are practicing dental implantology [22]. Our study showed that a large proportion of practitioners (91.2%) agreed with the need for increasing knowledge and awareness regarding the potential risk factors. The same study also showed that regular assessment of the theoretical and practical knowledge of implant dentistry is mandatory to improve their implant experience [22]. However, in our study, the majority (58%) considered dental education programs and workshops as the best way to achieve it while 40.3% considered regular assessment of the theoretical and practical knowledge of implants as the best way to achieve the aimed results. A study that was published by Tettamanti L et al. demonstrated that immediate loading implants showed a greater risk for implant failure when compared to conventional loading implants although the survival rates were high for both procedures [23]. Most practitioners (90.6%) agreed that immediate-loading implants have a greater risk of implant failure compared to conventional loading implants. Another study that was conducted by Momand P et al. concluded that antibiotic prophylaxis in conjunction with implant placement is likely of small benefit and thus should be avoided in most cases, especially given the unabated growth in antibiotic‐resistant bacteria [24]. Our study showed that the majority of practitioners (80.7%) usually prescribe a prophylactic antibiotic for dental implants. A study by Melini M illustrated that analgesic use seemed to be associated with improved postoperative outcomes (pain, patient satisfaction, and need for rescue medication) when compared to placebo [25]. In correlation, most practitioners (95.6%) support the importance of using analgesics and the association between patient satisfaction and pain management with the prescription of these analgesics. 

## Conclusions

All the targeted specialties exhibited a comparable impression regarding implant techniques and their outcomes. Each specialty had its own aspect of treatment during the placement of dental implants depending on multiple factors. Significant knowledge was observed from each specialty regarding implant brands, techniques, and antibiotic prescriptions for dental implants. Different training schools could be the reason for different implant favorable approaches. Moreover, each technique has its own considerations, depending on multiple factors such as medical history, bone status, implant cost, and patient motivation.

## References

[1] Do TA, Le HS, Shen YW, Huang HL, Fuh LJ (2020). Risk factors related to late failure of dental implant—a systematic review of recent studies. Int J Environ Res Public Health.

[2] Gupta R, Gupta N, Weber KK, DS DS (2021). Dental Implants. https://pubmed.ncbi.nlm.nih.gov/29262027/.

[3] Barreiros P, Neves L, Aroso C, Mendes JM, Silva AS (2020). Comparison in four different implant systems of mechanical resistance to maximal stress in prosthetic screws—an in vitro study. Dent J (Basel).

[4] Bassir SH, El Kholy K, Chen CY, Lee KH, Intini G (2019). Outcome of early dental implant placement versus other dental implant placement protocols: a systematic review and meta-analysis. J Periodontol.

[5] Seyssens L, De Lat L, Cosyn J (2021). Immediate implant placement with or without connective tissue graft: a systematic review and meta-analysis. J Clin Periodontol.

[6] Hamed MT, Mously HA, Ghulman MM, Naguib GH (2021). Impact of dental implant diameter on the efficiency of fatigue: a systematic review analysis. J Pak Med Assoc.

[7] Chrcanovic BR, Kisch J, Albrektsson T, Wennerberg A (2017). Impact of different surgeons on dental implant failure. Int J Prosthodont.

[8] Chappuis V, Avila-Ortiz G, Araújo MG, Monje A (2018). Medication-related dental implant failure: systematic review and meta-analysis. Clin Oral Implants Res.

[9] Abdelhay N, Prasad S, Gibson MP (2021). Failure rates associated with guided versus non-guided dental implant placement: a systematic review and meta-analysis. BDJ Open.

[10] Graf T, Keul C, Wismeijer D, Güth JF (2021). Time and costs related to computer-assisted versus non-computer-assisted implant planning and surgery. A systematic review. Clin Oral Implants Res.

[11] Hsu Y-T, Huang N-C, Wang H-L (2015). Relationship between periodontics and prosthodontics: the two-way street. Int J Periodontol Implantol.

[12] Tavelli L, Barootchi S, Avila-Ortiz G, Urban IA, Giannobile WV, Wang HL (2021). Peri-implant soft tissue phenotype modification and its impact on peri-implant health: a systematic review and network meta-analysis. J Periodontol.

[13] Sawase T, Kuroshima S (2020). The current clinical relevancy of intraoral scanners in implant dentistry. Dent Mater J.

[14] In 't Veld M, Schulten EA, Leusink FK (2021). Immediate dental implant placement and restoration in the edentulous mandible in head and neck cancer patients: a systematic review and meta-analysis. Curr Opin Otolaryngol Head Neck Surg.

[15] Velasco Bohórquez P, Rucco R, Zubizarreta-Macho Á (2021). Failure rate, marginal bone loss, and pink esthetic with socket-shield technique for immediate dental implant placement in the esthetic zone. A systematic review and meta-analysis. Biology (Basel).

[16] Lim HC, Seo S, Thoma DS, Park JC, Hong JY, Shin SY (2020). Late implant placement following ridge preservation versus early implant placement: a pilot randomized clinical trial for periodontally compromised non-molar extraction sites. J Clin Periodontol.

[17] Esposito M, Grusovin MG, Loli V, Coulthard P, Worthington HV (2010). Does antibiotic prophylaxis at implant placement decrease early implant failures? A Cochrane systematic review. Eur J Oral Implantol.

[18] Roca-Millan E, Estrugo-Devesa A, Merlos A, Jané-Salas E, Vinuesa T, López-López J (2021). Systemic antibiotic prophylaxis to reduce early implant failure: a systematic review and meta-analysis. Antibiotics (Basel).

[19] Gunes N, Gul M, Dundar S (2021). Biomechanical evaluation of implant osseointegration after guided bone regeneration with different bone grafts. J Craniofac Surg.

[20] Singh G, Pareek R, Rajawat GS, Kadam A, Al Abdulsalam M, Al Abdulathim A (2021). Comparison of bone healing in immediate implant placement versus delayed implant placement. J Pharm Bioallied Sci.

[21] Jalaluddin M, Sathe S, Thomas J, Haleem S, Naik S, Shivanna MM (2021). Assessment of implant stability in immediate implant placement using different bone grafting materials: a clinical study. J Pharm Bioallied Sci.

[22] Dutta SR, Passi D, Singh P, Atri M, Mohan S, Sharma A (2020). Risks and complications associated with dental implant failure: critical update. Natl J Maxillofac Surg.

[23] Tettamanti L, Andrisani C, Bassi MA, Vinci R, Silvestre-Rangil J, Tagliabue A (2017). Immediate loading implants: review of the critical aspects. Oral Implantol (Rome).

[24] Momand P, Becktor JP, Naimi-Akbar A, Tobin G, Götrick B (2022). Effect of antibiotic prophylaxis in dental implant surgery: a multicenter placebo-controlled double-blinded randomized clinical trial. Clin Implant Dent Relat Res.

[25] Melini M, Forni A, Cavallin F, Parotto M, Zanette G (2020). Analgesics for dental implants: a systematic review. Front Pharmacol.

